# Molecular Mechanisms of the Floral Biology of *Jatropha curca*s: Opportunities and Challenges as an Energy Crop

**DOI:** 10.3389/fpls.2020.00609

**Published:** 2020-06-09

**Authors:** Manali Gangwar, Jata Shankar

**Affiliations:** Genomic Laboratory, Department of Biotechnology and Bioinformatics, Jaypee University of Information Technology, Waknaghat, India

**Keywords:** *Jatropha curcas*, energy crop, transcriptome, biofuel, ABCDE model

## Abstract

Fossil fuel sources are a limited resource and could eventually be depleted. Biofuels have emerged as a renewable alternative to fossil fuels. *Jatropha* has grown in significance as a potential bioenergy crop due to its high content of seed oil. However, *Jatropha*’s lack of high-yielding seed genotypes limits its potential use for biofuel production. The main cause of lower seed yield is the low female to male flower ratio (1:25–10), which affects the total amount of seeds produced per plant. Here, we review the genetic factors responsible for floral transitions, floral organ development, and regulated gene products in *Jatropha*. We also summarize potential gene targets to increase seed production and discuss challenges ahead.

## Introduction

About 11 billion tons of oil is consumed worldwide each year for fuel. With this rate of oil consumption, we may soon exhaust the oil reservoir (Shafiee and Topal, 2009)^2^. Climate change is also greatly influenced by fossil fuel combustion. Therefore, sustainable and environmentally friendly alternative energy sources are needed. *Jatropha curcas* L (Euphorbiaceae) is a plant with potential for biodiesel production due to its high seed oil content (around 45–50%) (Achten et al., 2008). Compared with other oil plants, *Jatropha* has its own merits, including an outstanding adaptability to varied environments, smooth propagation, and greater fruit and seed size. Furthermore, *Jatropha* grows well in the desert, adapts to drought conditions, has a short gestation period, and assists in soil conservation. Despite its advantageous properties for biodiesel production, *Jatropha* has some limitations that restrict its commercialization as an energy crop, such as low seed yield, inconsistent flowering and fruiting, and relatively expensive plantation maintenance. The significant factors influencing its potential as biofuel feedstock are the oil content in seeds, the number of seeds per tree, the number of fruits on each branch of the plant, and the number of branches per plant. Seed yield at each inflorescence is largely dependent on the number of female flowers. *Jatropha*’s female to male flower ratio is quite small (1:25 to 1:30), which means that each inflorescence contains only about 10 to 12 female flowers (out of 300) that yield just 8 to 10 fruits. Therefore, a relatively small number of fruits are produced as compared to the total number of flowers (Kumar and Sharma, 2008). One way to increase the total seed yield in *Jatropha* would be to increase the number of female flowers per plant. In this context, we have discussed the genetic factors involved in the floral transformation, determination of sex, and floral growth of *Jatropha curcas*.

## Biology of Sex Determination in *Jatropha Curcas*

**FIGURE 1:**
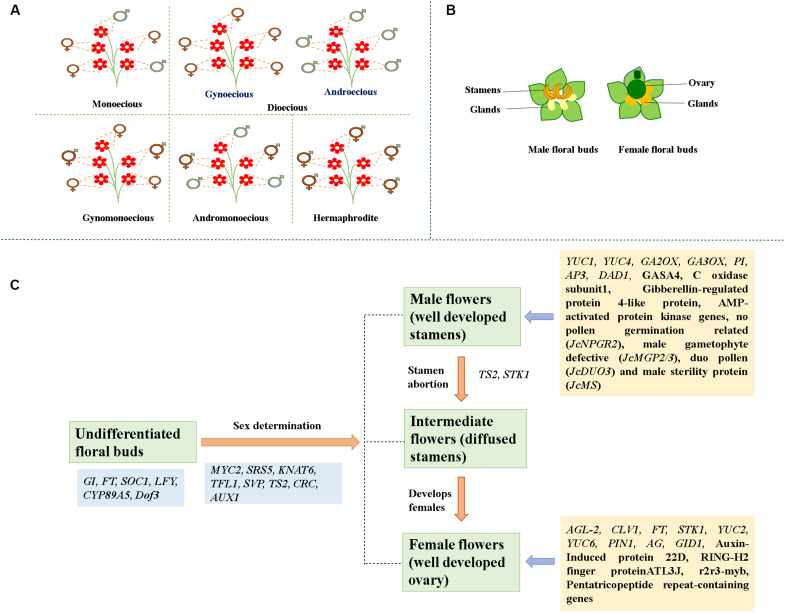
**(A)** Diversified morphology of flowers. 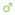 Represents male flowers; 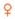 Represents female flowers; 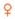 Represents hermaphrodite flowers. **(B)** Morphology of male and female floral buds in *Jatropha.* **(C)** Key genes involved in the floral transition, sex determination, and reproductive organ development (*GI-GIGANTEA; FT-FLOWERING LOCUS T; SOC1-SUPPRESSOR OF OVEREXPRESSION OF CONSTANS 1; LFY-LEAFY; CYP89A5-CYTOCHROME P450; SRS5-SHI-RELATED SEQUENCE 5; KNAT6-KNOTTED1-LIKE HOMEOBOX GENE 6; TFL1-TERMINAL FLOWER 1; SVP-SHORT VEGETATIVE PHASE; TS2-TASSELSEED2; CRC-CRABS CLAW; AUX1*-Auxin transporter protein 1; *STK1-SEEDSTICK; YUC1*-Flavin-containing monooxygenase; *YUC2/4/6*-Indole-3-pyruvate monooxygenase; *GA3ox*-Gibberellin 3-beta-hydroxylase; *GA20ox*-Gibberellin 20-oxidase; *PI-PISTILLATA; AP3-APETALA3; DAD1-DEFECTIVE IN ANTHER DEHISCENCE 1; GASA4*-Gibberellin-regulated protein 4 precursor; *AGL2-AGAMOUS-LIKE 2; CLV1-CALVATA1; PIN1-PIN-FORMED 1; AG2-AGAMOUS*-like protein 2; *GID1*-Gibberellin receptor protein).

Sex determination processes allow floral organ development in plants. The two processes for forming a unisexual flower are (i) emergence of only one type of sex organ (unisexual tissues) and (ii) initiation of stamen and pistil followed by an arrest or abortion of one sex organ, which results in the functional immaturity of either stamens or carpels. The developmental arrest step occurs at an immature stage well before sexual maturity is reached (Ainsworth, 1999, 2000; Kater et al., 2001). There are two modes of sex determination and development in *Jatropha*. One mode is the development of male flowers with early adolescence without any female primordia. The other mode is by aborting male tissues, which results in female flowers developing (Li and Li, 2009; Wu et al., 2011). The male flower is unisexual right from the start, whereas the female flower is bisexual until its sixth developmental phase. Because of this, an inflorescence has three types of flowering sites; (i) female flowering site, (ii) male flowering site, and (iii) middle flowering site where both males and females may develop. Though male tissue abortion occurs in female flowers during sexual differentiation, traces of male tissue may be found in mature females. However, when abortion of male tissues fails in a female flower, it develops as a male at the female flowering site. Such inflorescence is known as middle type inflorescence. Due to the number of female flowers formed at middle type inflorescence, variation in the total number of female flowers occurs. An inflorescence statistical analysis found ∼75 percent of middle-type inflorescence and 0.09 percent of female flowers (Luo et al., 2007; Wu et al., 2011). For 18 female locations, Wu et al. (2011) found only seven female flowers. The female flowering sites and the sites occupied by middle-type inflorescence are important in increasing the number of female flowers. The presence of hermaphroditic flowers has also been recorded in *Jatropha*, showing structural similarity with female flowers but diffused stamens (Abdelgadir et al., 2010; Wu et al., 2011; Adriano-Anaya et al., 2016). A recent population analysis on *Jatropha*’s floral diversity and sex expression has grouped accessions into gynoecious (having only females), androecious (having only males flowers), and andromonoecious (having both bisexual and male flowers) plants showing no correlation with their geographic location (Adriano-Anaya et al., 2016). Of the 103 accessions from 33 sites in southern Mexico, 93.2 percent were monoecious, while others were androgynomonoecious, androecious, or gynoecious (Figures 1A,B). It has been hypothesized that male development commences through suppression of females, which might be the result of male sterility mutation in gynomonoecious plants (Salvador-Figueroa et al., 2015; Adriano-Anaya et al., 2016). No gynomonoecious plants of *Jatropha* have been found. The possible explanation, according to Adriano-Anaya et al. (2016), is that gynoecious *Jatropha* plants derive from hermaphrodite ancestors through a one-step mutation.

## Genetic Factors for Vegetative to a Reproductive Phase Transition

In floral initiation, the apical shoot meristem differentiates into an inflorescence. The induction of floral signaling is genetically controlled by floral integrator genes, such as *FT* (*FLOWERING LOCUS T*), *FLC* (*FLOWERING LOCUS C*) and *SOC1* (*SUPPRESSOR OF OVEREXPRESSION OF CONSTANS 1*). Ye et al. (2014) reported that *JcFT* (*Jatropha Flowering locus T)* overexpression caused early flowering by shortening the bolting time. Li et al. (2014) characterized *FT* in *Jatropha*, and data from its spatial expression showed higher expression in reproductive phases. The *LFY* gene has recently been identified and overexpressed in both *Arabidopsis thaliana* and *Jatropha* (Tang et al., 2016). During the early stages of flowering, they observed a higher expression of *JcLFY* (*Jatropha LEAFY*). Transgenics with *JcLFY* overexpression showed early flowering and increased transcript levels of floral meristem identity genes, such as *JcAP1*, *JcAP3*, *JcSEP1*, *JcSEP3*, and *JcAG*. In addition, co-suppression of *LFY* in *Jatropha* resulted in delayed flowering, abnormal floral flowers replaced by sepaloid organs, and an increased rate of floral abortion (Tang et al., 2016). Recently, the role of *TFL1* homologs has been studied through the transgenic method, and their overexpression has resulted in delayed flowering due to reduced *AP1* and *FT* gene expression (Karlgren et al., 2013; Li et al., 2017). In contrast, Li et al. (2014) reported higher expression of *FT* in *Jatropha*’s reproductive phases and fruits. Circadian rhythms play an important role in the initiation of flowering. JcDof3, a plant-specific transcription factor with a conserved zinc finger (ZF) DNA-binding domain, is a circadian clock regulated gene. The C-terminal conserved region of Dof3 interacts with the F-box protein forming Dof3-Fbox complex regulating the expression of *CO*, a circadian clock regulating flowering gene (Yang et al., 2011). Foliar cytokinin (CTK) treatment upregulates genes *GI*, *SOC1*, and *LFY*, and inactivates genes *COP1* and *TFL1b* that maintain a flowering signal which promotes flowering (Chen et al., 2014; Pan et al., 2014). Thus, the interplay between the circadian rhythm and hormones control flowering genes and phase transition to inflorescence meristem in *Jatropha*.

## Molecular Basis of Sex Determination

*Jatropha* is a monoecious plant in which female flowers are formed due to stamen abortion/suppression. Remains of female tissues are not observed in male flowers, though remains of aborted stamens (male tissues) are present at the base in female flowers. By analyzing *Jatropha* floral buds for gene expression, the *SUPERMAN* gene was observed to suppress male tissue and promote the development of female tissue (Gangwar et al., 2018). A recent study suggested that, in *Arabidopsis*, the *SUPERMAN* gene not only bridges floral organogenesis and floral meristem but also regulates auxin biosynthesis (Xu et al., 2018). Transcriptome analysis of *Jatropha*’s floral buds showed reduced expression of the stamen development gene *TASSELSEED 2* (*TS2*) that facilitated the growth of carpels (Chen et al., 2014). Transcriptomic analysis of different stages of male and female flower buds of *Jatropha* showed upregulation of *CRABS CLAW* (*CRC*) during development stages of female flowers. *CRC*, a C2C2-YABBY zinc finger protein, is involved in the regulation of carpel fusion and growth, nectary formation, and floral meristem termination (Xu et al., 2016; Gross et al., 2018). Genes encoding for inorganic phosphate transporter and ubiquitin carboxyl-terminal hydrolase were upregulated during female flower development and may contribute to embryo sac development (Xu et al., 2016). Further, upregulation of genes encoding for chlorophyll A/B-binding protein during initiation of carpel primordia may facilitate carpel differentiation. Genes encoding for Gibberellin-regulated protein 4-like protein, cytochrome c-oxidase subunit 1 (mitochondrial gene), and AMP-activated protein kinase, however, were upregulated during stamen development. Upregulation of genes encoding for RING-H2 finger protein ATL3J (E3 ubiquitin ligases), *CLAVATA1* (receptor-like kinase), auxin-induced protein 22D, transcription factor R2R3-myb (regulating cell cycle genes and cytokinin signaling), and *AGAMOUS-LIKE-20* (MADS-box genes) have been identified during the late stage of female flower development, which may facilitate the maturation of female flower (Alvarez and Smyth, 1999; Pelaz et al., 2000; Makkena et al., 2012; Xu et al., 2016). In both male and female flower buds, genes such as ARP1 (Auxin repressed protein), X10A (auxin-induced protein), and GID1 (gibberellin receptor protein) were upregulated (Xu et al., 2016). The role of *JcFT*, a florigen and a key flowering pathway regulator in *Jatropha* showed significantly high transcript levels in female flowers (Li et al., 2014). Another transcriptomic study identified *MYC2*, *TS2*, *KNAT6*, *SVP*, *TFL1*, and *SRS5* as sex determination regulators in *J. curcas* (Chen et al., 2017). The suppression of nodulin *MtN3* or *LESS ADHERENT POLLEN* (*LAP3*) resulted in small anthers, sterile pollens, and abortion of female flowers in *Oryza sativa*, *Vitis vinifera*, and *Medicago truncatula* (Chu et al., 2006; Ramos et al., 2014). In *Pisum sativum L*, carpel senescence has been induced as a result of increased lipoxygenase gene expression (Rodríguez-Concepción and Beltrán, 1995). Pentatricopeptide repeat-containing gene is expressed in the female embryo sac and restores the cytoplasmic male sterility in *Jatropha* (Bentolila et al., 2002; Xu et al., 2016). Key genes involved in the floral transition, sex determination and development of reproductive organs are shown in Figure 1C and Table 1. These studies shed light on how sex determination and differentiation occur in monoecious plants and how some of the genes expressed during floral differentiation suppress male flowering.

**TABLE 1:** Key genes involved in the floral transition, sex determination, and reproductive organ development.

**Vegetative to reproductive stage**			
**Gene name**	**Plant spp.**	**Pathway/Association**	**References**
*1-aminocyclopropane-1-carboxylate synthase* (*ACS1*)	*Cucumis sativus*	Ethylene biosynthesis	Switzenberg et al., 2014
*1-aminocyclopropane-1-carboxylate synthase 7* (*ACS7*)	*Cucumis sativus*	Ethylene biosynthesis	Switzenberg et al., 2014
*Agamous* (*AG*)	*Populus trichocarpa*	MADS-box regulators of differentiation, Homeotic genes	Brunner et al., 2000
*Apetala 1/3* (*AP1/3*)	*Arabidopsis thaliana*	Floral meristem identity genes	Karlgren et al., 2013
Dof3	*Arabidopsis thaliana*	F-box protein regulates flowering time	Imaizumi, 2010
*Flowering locus C* (*FLC*)	*Arabidopsis thaliana*	Florigen signaling	Li et al., 2014
*Flowering Locus T* (*FT*)	*Arabidopsis thaliana*	Florigen signaling	Li et al., 2014
*GIGANTEA* (*GI*)	*Arabidopsis thaliana*	Circadian clock control and photoperiodism	Mizoguchi et al., 2005
*LFY*	*Arabidopsis thaliana*	Florigen signaling	Tang et al., 2016
*Sepallata* (*SEP1/3*)	*Arabidopsis thaliana*	Floral meristem identity genes	Pelaz et al., 2000
*Suppressor of constans overexpression 1 (SOC1)*	*Arabidopsis thaliana*	MADS-box protein	Chen et al., 2014

**Genes associated with flowering sex determination**			
*Aborted microspores* (*AMS*)	*Arabidopsis thaliana; Capsicum annuum* L.	Pollen and anther development	Ye et al., 2010; Guo et al., 2018
*Agamous* (*AG2*)	*Elaeis guineensis*	Ovule primordia and carpel	Favaro et al., 2003; Adam et al., 2007
*Agamous-like-2* (*AGL-*2)	*Arabidopsis thaliana*	Induces microsporogenesis, Embryo sac development	Pelaz et al., 2000
*Apetala 3* (*AP3*)	*Elaeis guineensis*	Development of male tissues	Favaro et al., 2003; Adam et al., 2007
*ARF 10/16/17*/*18*	*Arabidopsis thaliana*	Female organ abortion	Huang et al., 2016
ATL3J	*Zea mays*	Embryo sac development	Xu et al., 2016
Auxin induced protein (*X10A*)	*Arabidopsis thaliana*	Stamen differentiation and embryo sac development	Xu et al., 2016
Auxin repressed protein (*ARP1*)	*Nicotiana tabacum*	Pollen maturation	Nakamura et al., 2004; Xu et al., 2016
Clavata1 (*CLV1*)	*Arabidopsis thaliana*	Peptide-receptor signaling	Alvarez and Smyth, 1999
CRABS CLAW (*CRC*)	*Arabidopsis thaliana; Oryza sativa*	Carpel fusion and growth, forming nectary	Gross et al., 2018
*Cup-shaped cotyledon* 2 (*CUC2)*	*Arabidopsis thaliana; Silene latifolia*	Forms boundary between the organs and separates organs with meristem	Li et al., 2010
*Defective in Tapetal development and function 1* (*TDF1*)	*Arabidopsis thaliana*	Pollen and anther development	Zhu et al., 2008
Duo pollen (*DUO3*)	*Arabidopsis thaliana*	Regulator of Male Germline and embryogenesis	Brownfield et al., 2009
Floral binding protein 11 (*FBP11*)	*Cucumis sativus*	Ovule formation	Favaro et al., 2003
Gibberellin receptor protein (*GID1*)	*Oryza sativa*	Stamen differentiation and embryo sac development	Ueguchi-Tanaka et al., 2007; Xu et al., 2016
*Less adherent pollen* (*LAP3*)	*Oryza sativa*, *Vitis vinifera, Medicago truncatula*	Pollen development	Chu et al., 2006; Ramos et al., 2014
*Lonely guy* (*LOG*)	*Oryza sativa*	Maintains floral meristem activity and ovule development	Yamaki et al., 2011
*Male gametophyte defective* (*MGP2/3*)	*Arabidopsis thaliana*	Pollen tube growth and pollen germination	Deng et al., 2010
No pollen germination related (NPGR2)	*Arabidopsis thaliana*	A calmodulin-binding protein regulated pollen germination	Golovkin and Reddy, 2003
*Pistillata* (*PI*)	*Elaeis guineensis*	Development of male tissues	Favaro et al., 2003; Adam et al., 2007
*Seedstick1* (*STKI*)	*Arabidopsis thaliana*	Carpel development	Pinyopich et al., 2003
*Sporocyteless/nozzle (SPL/NZZ)*	*Arabidopsis thaliana*	Regulates anther cell differentiation	Liu et al., 2009
*Superman (SUP)*	*Arabidopsis thaliana*	Suppresses stamen development	Prunet et al., 2017
*Tasselseed 2* (*TS2*)	*Zea mays*	Stamen development by Pistil abortion	Acosta et al., 2009
*YUCCA* (*YUC1/2/4/6*)	*Arabidopsis thaliana*	Stamen development	Stepanova et al., 2011

**ABCDE Model Genes**			
*Pistillata* (*PI*)	*Elaeis guineensis; Jatropha curcas*	B-class, stamen development	Favaro et al., 2003; Adam et al., 2007; Hui et al., 2017

**ABCDE Model Genes**			
**Gene name**	**Plant spp.**	**Pathway/Association**	**References**

*Apetala 3* (*AP3*)	*Elaeis guineensis; Jatropha curcas*	B-class, stamen development	Favaro et al., 2003; Adam et al., 2007; Hui et al., 2017
*Agamous* (*AG*)	*Jatropha curcas; Populus trichocarpa*	C-class, carpel differentiation	Brunner et al., 2000; Hui et al., 2017
*SEEDSTICK1* (*STKI*)	*Arabidopsis thaliana; Jatropha curcas*	D-class, carpel maturation	Pinyopich et al., 2003; Hui et al., 2017
*Sepallata* (*SEP*)	*Arabidopsis thaliana; Jatropha curcas*	E-class, male floral initiation	Pelaz et al., 2000; Chen et al., 2019

## Abcde Model for Sex Differentiation

The ABCDE model is a scientific model that specifies the role of homeotic genes in the development of floral organs. Genes of the A class specify sepal development. The development of petals occurs by the combined effect of genes from the A and B classes. Both the B- and C-class genes are important for stamen growth. The carpel development and activity of ovules are determined by C-class and D-class genes, respectively. Recently, E-class genes were discovered to play a role in the development of carpel and ovary (Pelaz et al., 2000; Honma and Goto, 2001). A-, B-, C-, D-, and E-class genes are transcription factors with conserved DNA binding domains known as the MADS-box family and are involved in floral organogenesis regulation (Parěnicová et al., 2003; Chen et al., 2019). *PERIANTHA* (*PAN*), a bZIP transcription factor, activates *AG*, a C-class MADS-box protein that regulates floral organ numbers and whorl patterning (Maier et al., 2009). In *Elaeis guineensis*, the mutants *AP3* and *PISTILLATA* (*PI*) inhibited male tissues. *AG2* has a mixed C/D function gene, and its expression has been observed in ovule primordia and carpel of *Arabidopsis* and *Elaeis guineensis*, respectively (Favaro et al., 2003; Adam et al., 2007). FLORAL BINDING PROTEIN 11 (FBP11), a D-class gene, determines the formation of ovules in cucumbers (Favaro et al., 2003). An increase in the C-class gene transcription level arrests the development of sexual organs in monoecious plants, such as *Liquidambar styraciflua L* and *Rumex acetosa L* (Ainsworth, 2000). B-and C-class genes are regulated at a sex locus by a genetic switch that further controls the development of male or female flowers in *Populus trichocarpa* (Leseberg et al., 2006). B-class genes *PI* and *AP3* have been identified in the formation of stamen in *Jatropha*. A- and C-class gene *AG* and D-class gene *SEEDSTICK1* (*STKI*) have been reported for carpel development and maturation (Hui et al., 2017). Thus, the ABCDE model helps to understand the floral differentiation in *Jatropha*.

## Role of Hormones in Sex Determination

The process of flower development and sex determination is regulated by the interplay of endogenous hormones (auxins, cytokinins, gibberellins, abscisic acids, etc.). Auxin regulates sex determination in *Jatropha*. IAA enhanced female to male ratio from 1:27 to 1:23, and it also increased seed weight 3-fold (Joshi et al., 2011). Auxin biosynthesis and signaling are associated with genes such as *ARFs*, *AUX1*, and Transport inhibitor response 1 (*TIR1*). Transcriptome analysis of *Jatropha* suggested that *AUX1* is responsible for sex determination. The main source of auxin production is through Trp-dependent auxin biosynthesis, which participates in embryo patterning and reproductive organ development (Chen et al., 2017). In this pathway, IAA is produced from indole-3-pyruvic acid by *YUCCA* (*YUC*), a flavin-dependent monooxygenase (Stepanova et al., 2008). During stamen primordia formation, auxin is produced locally by *YUC1* and *YUC4* followed by *YUC2* and *YUC6* genes at late stages of stamen development (Cheng et al., 2006; Cecchetti et al., 2008). In mature gynoecia, *YUC4* and *YUC8* genes were expressed in the style, whereas *YUC2* was expressed in carpel valve tissues (Martínez-Fernández et al., 2014). Increased expression of *ARF 10/16/17/18* leads to abnormalities in females and abortion of organs, resulting in fewer seed sets (Huang et al., 2016).

Gibberellic acids also contribute to the development of the stamens in monoecious plants. Exogenous application of GA on the inflorescences of *Jatropha* resulted in a 2-fold increase in female flowering. However, inflorescence branches were not affected. Hui et al. (2018) reported the altered endogenous CTK (increased) and GA (decreased) ratio due to exogenous GA application, which resulted in an increased proportion of female flowers. However, a higher concentration of GA caused withering of floral buds. Hu et al. (2017) isolated the *JcGA2ox6* (Gibberellin oxidase) gene, which reduces the amount of endogenous GA4 (active gibberellin). They overexpressed *JcGA2ox6* gene in *Jatropha*, which led to decreased inflorescence size, decreased male and female flowers, and decreased seed length in transgenic plants. There was a significant decrease in both seed weight and oil content. *GA20ox* and *GA3ox* have been observed in other studies to enhance the development of stamen, whereas the exogenous application of GA3 led to a restricted development of pistils, thus enabling the male to expand. GA treatment enhanced the development of stamens in monoecious females, and it resulted in bisexual flowers in monoecious plants. GASA4 protein functions in stamen differentiation. *GID1*, a positive GA signaling pathway regulator, controls *Jatropha*’s female flowering (Roxrud et al., 2007; Hu et al., 2017). GA deficiency results in male sterility in plants. Therefore, GA allows the stamens to develop without affecting female flowers.

Paclobutrazol foliar application inhibits GA biosynthesis and promotes female flowering by suppressing no related pollen germination (*JcNPGR2*), male defective gametophyte (*JcMGP2*/3), duo pollen (*JcDUO3*), and male sterility protein (JcMS) genes, thus allowing female flowers to develop in *Jatropha* (Seesangboon et al., 2018).

Jasmonic acids and brassinosteroids (BRs) are active in floral development together with stamen development, pollen maturation, and male fertility (Park et al., 2002; Ye et al., 2010). In staminate maize flowers, brassinosteroids promoted pistil abortion. AG controls the maturation and late stages of stamen development in *Arabidopsis* by regulating the biosynthesis of jasmonates (Ito et al., 2007). Reduced JA synthesis in *Jatropha* led to male abortion and downregulation of the genes *DAD1* and *LOX2*. *Arabidopsis*, maize, and tomato mutants with suppressed jasmonate synthesis and brassinosteroid signaling resulted in male sterility (Li et al., 2005; Ye et al., 2010). The *SPL*/*NZZ*, *Aborted Microspores* (*AMS*) and D*efective in Tapetal Development and Function 1* (*TDF1*) genes are regulated by BRs and are critical for anther and pollen development (Ye et al., 2010). Thus, BRs and JAs promote the development of male organs.

Foliar application of ethylene induced femininity in *Jatropha*. To synthesize ethylene, 1-aminocyclopropane-1-carboxylic acid oxidase 2 (*ACO1*) oxidizes ethylene intermediates. Transgenics plants that overexpressed *ACO2* were male sterile due to suppressed stamens. Little to no activity of *ACO* was observed in *Arabidopsis*, tomato, and tobacco during the development of anthers and pollens (Bartley and Ishida, 2007; Duan et al., 2008; Wang et al., 2010). These experiments have thus shown that ethylene promotes feminism in plants.

Studies have been conducted to see the effect of foliar cytokinin application on the inflorescences. It has been found that 29.99 percent of the total flowers were females in treated inflorescences as compared to 6.96 percent in control. In treated inflorescence, a 4–5-fold increase in the number of seeds was observed but the fruiting rate, seed weight, and oil content decreased (Pan and Xu, 2011; Pan et al., 2014; Chen et al., 2014).

Transcriptomic analysis of *Jatropha* inflorescences treated with cytokinin revealed that genes involved in the initiation of flowers, such as *GI*, *SOC1*, and *LFY*, and the *CYP89A5* gene involved in the development of inflorescences were induced, whereas the *AP1*, *AP2*, *PI*, *AG*, and *SEP1-3* genes were downregulated (Chen et al., 2014; Pan et al., 2014). These developments allowed more time for inflorescence meristems to generate floral primordia. A vital increase in the number of flowers was noted due to *CUC1* upregulation. Application of BA (6-Benzylaminopurine) increased the rate of cell division in inflorescence meristem due to the upregulation of *Cyclin-3-1* (*CycD3;1/2*) and *Cyclin-dependent protein kinase* 247 (*CycA3;2*) genes. Li et al. (2010) observed an increase in the number of flowers with an enlarged inflorescence and floral meristem in transgenic *Arabidopsis* overexpressing CK (cytokinin) biosynthetic gene (*AtIPT4*). Fewer flowers were observed at each inflorescence due to the overexpression of the *CKX* gene (Werner and Schmülling, 2009). Loss-of-function mutation of *LONELY GUY* (*LOG*) (encodes for CK-activating enzyme) gene of rice led to the significant decrease in the number of floral organs (Kurakawa et al., 2007). Chen et al. (2014) reported that BA treatment decreased the expression of *TS2*, which suppresses carpel in maize, leading to increased female to male flower ratios in *Jatropha* (Acosta et al., 2009).

## Challenges

Genomic studies on flowering of *Jatropha* and phenotypic changes following the application of PGRs (Plant Growth Regulators) showed an opportunity to increase female flowering, which is one of the aspects for increasing seed yields. There are several challenges to increasing a number of female flowers: (i) manual hormone application to each inflorescence is laborious; (ii) hormone application is not economical; (iii) optimized hormone concentration at one environmental condition may not show the same efficiency under different environmental conditions; (iv) flowering and fruit maturity are not synchronized; and the (v) variation in fruiting rate. Genetic modification of flowering genes or overexpression of genes involved in suppression of male flowers may enable us to overcome these challenges by allowing more female flowers to develop. Other possibilities include enhancing cytokinin synthesis by overexpressing genes associated with cytokinin biosynthesis or suppressing cytokinin breakdown by gene silencing or mutagenesis. Additionally, further research could be carried out on the effect of central carbon flow on the fruiting rate.

## Conclusion and Perspective

The female to male floral ratio plays a significant role in deciding *Jatropha*’s seed yield. Cytokinin application showed promising results in enhancing the ratio between female and male flowers. Promising approaches to increase the number of female flowers may be to induce the transitioning of male type inflorescences to the middle/intermediate type or to increase male flower abortion rates to allow female flowers to develop. Therefore, genes involved in female flowering or the abortion of male flowers could be targeted for the purpose of increasing female flowers in *Jatropha*.

## Author Contributions

MG and JS conceived and designed the review manuscript, wrote, read, and approved the manuscript. JS contributed materials or analytical tools and supervised the work.

## Conflict of Interest

The authors declare that the research was conducted in the absence of any commercial or financial relationships that could be construed as a potential conflict of interest.

## Footnotes

1 https://www.ecotricity.co.uk/our-green-energy/energy-independence/the-end-of-fossil-fuels

## Abbreviations

BRs: brassinosteroids
CTK: cytokinin
GAs: gibberellic acids
JAs: jasmonic acid
*Jatropha*: *Jatropha curcas* L.
